# SLC30A10 downregulation is associated with cGAS-STING pathway activation in colorectal tubular adenoma

**DOI:** 10.1038/s41598-026-48815-6

**Published:** 2026-04-16

**Authors:** Tongshuo Qu, Guoju Jin, Liping Zhang, Tangyou Mao, Yupu Yao, Runhua Chen

**Affiliations:** 1 Department of Gastroenterology, Beijing University of Chinese Medicine Dongfang Hospital, No. 6 Fangxingyuan Area 1, Fangzhuang, Fengtai District, Beijing, 100078 China; 2 Department of Pathology, Beijing University of Chinese Medicine Dongfang Hospital, No. 6 Fangxingyuan Area 1, Fangzhuang, Fengtai District, Beijing, 100078 China; 3 Beijing University of Chinese Medicine, No. 11 Beisanhuan East Road, Chaoyang District, Beijing, 100029 China

**Keywords:** Colorectal tubular adenoma, SLC30A10, cGAS-STING pathway, DIA proteomics, Cancer, Diseases, Gastroenterology

## Abstract

Based on data-independent acquisition (DIA) proteomics technology, to analyze the proteomic characteristics of colorectal tubular adenoma and explore the expression changes of SLC30A10 and their potential association with the cGAS-STING pathway. A self-controlled design was adopted, collecting colorectal tubular adenoma (TA) and paired normal mucosa (NM) from 15 patients with TA. Differentially expressed proteins were screened by DIA proteomics, followed by GO and KEGG enrichment analyses. Immunohistochemistry was performed to detect the expression of SLC30A10, cGAS, STING, p-IRF3, ISG15, and β-catenin; immunofluorescence double staining was used to observe the co-localization of p-IRF3 and β-catenin; inductively coupled plasma mass spectrometry (ICP-MS) was employed to determine tissue manganese content. DIA analysis showed that SLC30A10 protein expression was significantly downregulated in TA tissues. Functional enrichment analysis indicated abnormalities in signal transduction, metabolic reprogramming, and nitrogen metabolism in TA tissues. IHC results demonstrated that, compared with NM, TA tissues exhibited reduced expression of SLC30A10, while the expression of cGAS, STING, p-IRF3, ISG15, and β-catenin was upregulated. Manganese content in TA tissues was also significantly increased. Immunofluorescence revealed enhanced nuclear signals of p-IRF3 in TA cells, with co-localization of p-IRF3 and β-catenin observed in the nucleus. Downregulation of SLC30A10 in colorectal tubular adenoma is associated with manganese accumulation and alterations in the cGAS-STING pathway, suggesting its potential role in the development and progression of adenoma, a finding with promising research implications.

**Supplementary Information:**

The online version contains supplementary material available at 10.1038/s41598-026-48815-6.

## Introduction

Colorectal tubular adenoma (TA), as the most common type of colorectal adenomatous polyp, is a significant precancerous lesion of colorectal cancer (CRC). Its risk of malignant transformation is closely associated with adenoma size (increased risk for diameter > 1 cm) and histological grade (pathologically diagnosed as high-grade dysplasia)^1^. Currently, endoscopic resection serves as the primary treatment; however, the 3-year recurrence rate for advanced adenomas remains as high as 19.8%^2^. The clinical manifestations of TA patients lack specificity, making the exploration of dynamic changes at the protein level particularly important for early identification of high-risk populations and refined risk stratification.

In recent years, Data-Independent Acquisition (DIA) quantitative proteomics technology has provided a powerful tool for protein research. Its characteristics enable high-precision, high-stability, and high-reproducibility quantitative analysis of proteins, including low-abundance ones, in clinical cohort samples, significantly enhancing the reliability and depth of disease biomarker screening and molecular mechanism research^3^.

Based on preliminary analysis using DIA technology, this study identified a significant downregulation of Solute Carrier Family 30 Member 10 (SLC30A10) in TA samples. SLC30A10 is a manganese efflux transporter, and its loss of expression has been confirmed by multi-center cohort studies (covering GEO datasets and TCGA database) to be positively correlated with CRC progression and metastasis^4^. More crucially, hypermethylation of the SLC30A10 promoter has been established as an early diagnostic marker for CRC, suggesting its upstream role in the temporal sequence of carcinogenesis^5^. The cGAS-STING pathway is a core innate immune signaling pathway within cells, where cGAS synthesizes the second messenger cGAMP to activate the STING protein, subsequently promoting the formation of p-IRF3 and initiating anti-tumor immune responses. Current research has demonstrated its close association with colorectal adenocarcinoma^6^. Based on this, we hypothesize that the downregulation of SLC30A10 may lead to an imbalance in intracellular manganese homeostasis, which may in turn be related to the activation of the cGAS-STING signaling pathway and play a role in the formation of adenomas. Therefore, our team collected intestinal adenoma tissues and normal mucosa from patients, compared whole proteome and differential protein data, performed GO and KEGG pathway annotation and enrichment analysis, and conducted experiments such as immunohistochemistry, immunofluorescence double staining, and tissue manganese content measurement to test the aforementioned mechanistic hypothesis, aiming to discover prognostic biomarkers and novel therapeutic targets for colorectal adenomas.

## Materials and methods

### General information

Data from patients who underwent electronic colonoscopy at the Endoscopy Center of Dongfang Hospital, Beijing University of Chinese Medicine, between July 1, 2025, and December 31, 2025, and were found to have colorectal polyps with pathological diagnosis of tubular adenoma, were included. This study adopted a self-controlled design, with a total of 15 patients enrolled. Inclusion criteria: ① Age 18–80 years; ② Colorectal polyps detected by colonoscopy and confirmed by pathological diagnosis; ③ Patients provided informed consent and signed the informed consent form. Exclusion criteria: ① Coexisting conditions such as intestinal malignancy or intestinal obstruction; ② Severe diseases of vital organs including heart, liver, or kidney; ③ Patients with mental disorders, impaired consciousness, or poor compliance. This study was approved by the Dongfang hospital’s Ethics Committee (Ethics Approval Number: 2025-05-02.01).

### Diagnostic criteria

The definition of colorectal adenoma was based on the *2024 ESGE Guideline on Colorectal Polypectomy and Endoscopic Mucosal Resection (EMR)*^1^: Colorectal adenomas are any protrusions from the colorectal mucosal surface, which may be neoplastic or non-neoplastic. Adenomatous polyps include tubular adenoma, villous adenoma, and mixed adenoma, carrying a risk of malignant transformation and requiring complete resection.

### Colonoscopy procedure and sample collection

Prior to colonoscopy, patients were instructed to undergo adequate bowel preparation, using polyethylene glycol (PEG) for bowel cleansing one day in advance. The Boston Bowel Preparation Scale score should reach Grade I or II. During the procedure, the endoscope was advanced from the rectum to the ileocecal valve, with comprehensive observation during withdrawal. Upon discovery of an adenoma, detailed histological information was recorded, and a biopsy was taken using biopsy forceps. Subsequently, normal mucosal tissue was randomly selected from an area approximately 5 cm away from the center of the adenoma and biopsied using forceps. Three samples each of tubular adenoma tissue and normal intestinal tissue were collected from each patient for DIA analysis, immunohistochemistry/immunofluorescence, and tissue manganese content measurement, respectively.

### Demographic information and sample grouping

A total of 15 samples each of tubular adenoma tissue and normal mucosal tissue were collected, designated as the Adenoma Group (Tubular Adenoma, TA) and the Normal Group (Normal Mucosa, NM), respectively. Demographic information and sample grouping details are shown in Table 1. The location, size, and number of adenomas per patient are listed in Table 2.

**Table 1:** Demographic information and sample grouping details.

ID	Gender	Age (years)	TA (tissue weight, mg)	NM(tissue weight, mg)
P1	Male	67	TA1 (10.3)	NM1 (10.4)
P2	Male	59	TA2 (10.1)	NM2 (10.0)
P3	Female	48	TA3 (9.8)	NM3 (10.3)
P4	Male	62	TA4 (10.5)	NM4 (9.8)
P5	Female	71	TA5 (10.7)	NM5 (9.9)
P6	Female	53	TA6 (9.6)	NM6 (10.4)
P7	Female	65	TA7 (10.6)	NM7 (10.0)
P8	Male	68	TA8 (11.0)	NM8 (11.0)
P9	Female	60	TA9 (9.7)	NM9 (10.3)
P10	Male	69	TA10 (9.6)	NM10 (10.4)
P11	Male	73	TA11 (10.4)	NM11 (10.7)
P12	Male	72	TA12 (10.6)	NM12 (9.7)
P13	Male	64	TA13 (10.0)	NM13 (10.3)
P14	Female	68	TA14 (10.2)	NM14 (10.5)
P15	Female	75	TA15 (10.3)	NM15 (10.3)

**Table 2:** Adenoma sample information.

Sample name	Adenoma location	Adenoma size (cm)	Adenoma number (*n*)
TA1	Ascending Colon	0.2*0.2	1
TA2	Transverse Colon	0.5*0.4	3
TA3	Sigmoid Colon	0.6*0.8	4
TA4	Sigmoid Colon	1.5*2.0	6
TA5	Ascending Colon	0.3*0.3	2
TA6	Transverse Colon	0.4*0.6	1
TA7	Sigmoid Colon	0.5*0.3	1
TA8	Sigmoid Colon	0.5*0.6	1
TA9	Descending Colon	1.0*0.8	3
TA10	Sigmoid Colon	0.3*0.5	2
TA11	Descending Colon	0.5*0.7	1
TA12	Descending Colon	0.4*0.3	2
TA13	Sigmoid Colon	0.3*0.5	1
TA14	Sigmoid Colon	0.3*0.5	3
TA15	Descending Colon	0.4*0.4	1

### Instruments and reagents

The instruments used in this study include: Vortex-Genie 2 vortex mixer (Sigma); ABS-MS-078 constant temperature mixer (Hefei Abson Scientific Instruments Co., Ltd.); Spectra Max plus384 microplate reader (Molecular Devices); Vanquish Neo high-performance liquid chromatography (HPLC) system, Orbitrap Astral mass spectrometer, NanoDrop One spectrophotometer (Thermo Fisher Scientific); tissue dehydrator, paraffin embedding machine, -20 °C freezing station, microtome, tissue spreading and baking machine (Leica); fluorescence microscope (OLYMPUS); ICP-MS (Agilent); microwave digestion system (Shanghai Yi Yao Instrument Technology Development Co., Ltd.), etc.

The reagents used in this study include: Triethylammonium bicarbonate (TEAB) buffer (Sigma); mass spectrometry-grade Modified Trypsin protease (Promega); Coomassie Brilliant Blue staining solution, rabbit anti-human β-catenin polyclonal antibody (AF0066) (Beyotime Biotechnology, Shanghai); Pierce BCA Protein Assay Kit, NuPAGE 10% BT GEL 1.0MM 12-well, Bond-Breaker TCEP solution, 5.5 cm High Throughput µPAC Neo HPLC Column, DAPI staining solution, rabbit anti-human ISG15 monoclonal antibody (1H9L21), ProLong Gold antifade mountant (Thermo Fisher Scientific); rabbit anti-human SLC30A10 polyclonal antibody (CSB-PA747695LA01HU, Wuhan Huamei Biological Engineering Co., Ltd.); rabbit anti-human STING polyclonal antibody (AF8073, Beyotime Biotechnology, Shanghai); rabbit anti-human p-IRF3 polyclonal antibody (IPDX22421, Hubei Aipti Bioengineering Co., Ltd.); rabbit anti-human cGAS polyclonal antibody (bs-9537R, Beijing Biosynthesis Biotechnology Co., Ltd.); HRP-conjugated goat anti-rabbit IgG (GB23303), Alexa Fluor 594-conjugated goat anti-rabbit IgG (GB28301), Alexa Fluor 488-conjugated goat anti-rabbit IgG (GB25303) (Wuhan Servicebio Technology Co., Ltd.), etc.

### Detection methods

#### Proteomics analysis

① Protein extraction: All samples were taken out under frozen conditions and transferred to lysis tubes. An appropriate volume of protein lysis buffer was added. Tissues were homogenized using a high-throughput tissue homogenizer with three rounds of vortexing. After non-contact low-temperature ultrasonication and centrifugation, the supernatant was collected. Protein concentration was determined using the BCA method, followed by SDS-PAGE electrophoresis.
② Protein digestion: 100 µg of protein sample was taken, supplemented with lysis buffer, and incubated at 37 °C for 60 min. Iodoacetamide was added to a final concentration of 40 mM and reacted at room temperature in the dark for 40 min. Pre-chilled acetone was added to each tube, and proteins were precipitated at -20 °C for 4 h. After centrifugation, the pellet was collected. The pellet was fully dissolved in TEAB buffer, and Modified Trypsin protease was added for overnight digestion at 37 °C.
③ Peptide desalting and quantification: After tryptic digestion, peptides were dried using a vacuum centrifugal concentrator and reconstituted. Desalting was performed using HLB solid-phase extraction (SPE) columns. Peptides were dried again using the vacuum concentrator, and peptide quantification was performed via UV spectrophotometry using the NanoDrop One spectrophotometer.
④ Mass Spectrometry Detection: Equal amounts of peptides were dissolved in mass spectrometry loading buffer for DIA analysis. Peptide separation was performed using the Vanquish Neo HPLC system. Mass spectrometry analysis was conducted using the Vanquish analytical purification HPLC system coupled with the mass spectrometer.
⑤ Data processing: Raw DIA data were imported into the Spectronaut 19 software for database searching analysis. The protein sequence database used in this study was sourced from Ensembl. A spectral library was constructed from the merged sample data using a combined threshold of precursor-level FDR ≤ 1% and protein-level FDR ≤ 1%.
⑥ Missing value filtering and imputation: The raw output data from Spectronaut 19 software underwent the following preprocessing steps: (a) Missing Value Filtering: Proteins with missing values exceeding 80% across all samples were removed to eliminate noise signals from low-abundance or random detection. (b) Missing Value Imputation: For the remaining proteins, if the proportion of non-missing samples within any group (TA or NM) reached 30% or more, the missing values for that protein in that group were considered potentially indicative of group-specific expression. For such missing values, imputation was performed using the k-nearest neighbors (KNN) algorithm separately for each group to maximally preserve biological information and reduce the impact of missing values on statistical analysis. Proteins with missing values that did not meet this condition were removed.

#### Immunohistochemistry (IHC) detection

① Tissues were fixed in formalin, dehydrated, and embedded in paraffin. Serial Sect.  (4 μm) were prepared from each tissue block for immunohistochemical staining of different markers. Sections were mounted on slides and baked. All sections were reviewed by a pathologist to confirm the adenoma regions and corresponding normal mucosa.
② Deparaffinization and Hydration: Sections were treated with xylene and a graded ethanol series, followed by circling with an IHC pen.
③ Antigen retrieval: Antigen retrieval was performed by microwave heating in EDTA solution, followed by slow cooling to room temperature.
④ Blocking: Endogenous peroxidase blocking was performed first, followed by the addition of QuickBlock immunostaining blocking solution.
⑤ Primary antibody incubation: Primary antibodies were diluted at appropriate ratios and incubated overnight at 4 °C.
⑥ Secondary antibody incubation: Secondary antibodies were added dropwise and incubated at room temperature for 60 min.
⑦ DAB staining and counterstaining: DAB working solution was added dropwise, observed under a microscope, rinsed with tap water, and then immersed in hematoxylin staining solution for counterstaining.
⑧ Dehydration and mounting: Sections were dehydrated through a graded ethanol series and xylene, then mounted with neutral balsam for observation.

#### Immunofluorescence double staining detection

① Sample preparation, deparaffinization, hydration, and antigen retrieval: See steps ①, ②, and ③ in "Immunohistochemistry (IHC) detection"
② Blocking: QuickBlock immunostaining blocking solution was added.
③ β-catenin staining:
  - Primary Antibody Incubation: Diluted β-catenin working solution was added dropwise, and slides were placed in a humidified chamber for overnight incubation at 4 °C.
  - Washing: The primary antibody was retrieved, and slides were washed 3 times (5 min each) with washing buffer.
  - Secondary antibody incubation: Alexa Fluor 594-labeled anti-rabbit secondary antibody, diluted in blocking buffer, was added dropwise and incubated in the dark at room temperature for 1 h, followed by 3 washes (5 min each) with washing buffer.
④ Antibody elution:
  - Eluting First-Round Antibodies: Immunostaining antibody elution solution was added dropwise onto the tissue.
  - Thorough Washing: Slides were thoroughly washed 3 times (5 min each) with washing buffer to completely remove the elution solution.

⑤ p-IRF3 Staining:
  - Re-blocking: Same as step ②.
  - Primary antibody incubation: Diluted p-IRF3 working solution was added dropwise, and slides were placed in a humidified chamber for overnight incubation at 4 °C.
  - Washing: The primary antibody was retrieved, and slides were washed 3 times (5 min each) with washing buffer.
  - Secondary antibody incubation: Alexa Fluor 488-labeled anti-rabbit secondary antibody, diluted in blocking buffer, was added dropwise and incubated in the dark at room temperature for 1 h, followed by 3 washes (5 min each) with washing buffer.

⑥ Nuclear staining and mounting:

- DAPI staining solution was added dropwise and incubated at room temperature for 5 min.
- Washing: Slides were washed 3 times (5 min each) with washing buffer in the dark.
- Mounting: ProLong Gold antifade mounting medium was added dropwise, coverslips were applied, and observation was performed using a fluorescence microscope as soon as possible.

#### Tissue manganese content detection

① Inductively Coupled Plasma Mass Spectrometry (ICP-MS) Instrument Operating Parameters: RF power 1550 W, plasma gas flow rate 15.0 L/min, carrier gas flow rate 0.8 L/min, auxiliary gas flow rate 0.40 L/min, helium gas flow rate 5 mL/min, nebulizer chamber temperature 2 °C.
② Sample preparation: Approximately 50 mg (wet weight) of colon tissue was weighed and placed into a microwave digestion vessel. 8 ml of nitric acid was added. The digestion program was set as follows: ramp to 120 °C in 5 min, then to 180 °C in 10 min, and hold at 190 °C for 25 min. Acid evaporation was performed at 110 °C for 6 h. The solution was then diluted to a final volume of 50 mL to obtain the test sample solution. A reagent blank test was performed simultaneously.
③ Preparation of manganese standard solution: 0.05 mL of manganese standard solution (1000 mg/L) was pipetted into a 50 mL volumetric flask. Nitric acid solution was slowly added and mixed to obtain a 1 mg/L intermediate stock solution. The intermediate stock solution was serially diluted to prepare standard series working solutions (concentrations: 0, 0.5, 1.0, 5.0, 10, 50, 100, 200, 500 µg/L).
④ Preparation of multi-element internal standard working solution: 0.5 mL of multi-element internal standard stock solution was pipetted into a 100 mL volumetric flask. Nitric acid solution was slowly added and mixed, and finally diluted to 500 µg/L. The multi-element internal standard solution included Sc, Ge, Rh, In, Re, Bi. The internal standard element used in this study was In.
⑤ Calculation of tissue manganese content:

$$\:\mathrm{M}\mathrm{a}\mathrm{n}\mathrm{g}\mathrm{a}\mathrm{n}\mathrm{e}\mathrm{s}\mathrm{e}\:\mathrm{c}\mathrm{o}\mathrm{n}\mathrm{t}\mathrm{e}\mathrm{n}\mathrm{t}\:({\upmu\:}\mathrm{g}/\mathrm{g})\:=\frac{(\mathrm{C}\:sample\text{}-\mathrm{C}\:blank\text{})\times\:\mathrm{V}}{\mathrm{W}}$$

C sample is the manganese concentration (µg/L) measured by ICP-MS in the sample solution; C blank is the manganese concentration (µg/L) measured by ICP-MS in the reagent blank solution from the same batch; V is the final sample volume (L); W is the wet weight of the colon tissue weighed (g).

### Statistical methods

#### IHC and immunofluorescence image processing

IHC-stained sections were observed and photographed using an optical microscope. Images were converted to grayscale using ImageJ software for semi-quantitative analysis to calculate the Average Optical Density (AOD) for SLC30A10, cGAS, STING, p-IRF3, ISG15, and β-catenin (AOD = IntDen / Area). β-catenin and p-IRF3 double-stained fluorescence sections were observed in a darkroom and photographed using a fluorescence microscope, and the average optical density was calculated.

#### Statistical analysis

##### Proteomics detection

Statistical analysis for proteomics was performed using R language (v4.4.3) and related packages. To assess experimental reproducibility and inter-group separation, Pearson correlation coefficients were calculated for all samples and a sample correlation heatmap was plotted. Intra-group coefficients of variation (CV) were calculated to evaluate quantitative precision. Based on the quantitative matrix after missing value filtering and imputation, to identify differentially expressed proteins (DEPs), a paired-sample t-test was performed for each protein to compare the expression levels between adenoma tissue and paired normal mucosa from the same patient. To control false positives arising from multiple hypothesis testing, the obtained p-values were adjusted using the Benjamini-Hochberg method for false discovery rate (FDR) correction, resulting in adjusted p-values (q-values). The screening criteria for DEPs were: proteins with a Fold Change (FC) > 1.2 (TA/NM) and q-value < 0.05 were considered up-regulated; proteins with FC < 0.83 and q-value < 0.05 were considered down-regulated. GO annotation and enrichment analysis were performed on the DEPs using the GO database. Signal pathway annotation and enrichment analysis were performed using the KEGG pathway database. Enrichment analysis employed the hypergeometric test, also adjusted using the Benjamini-Hochberg method for FDR correction, with a significance threshold set at q-value < 0.05.

##### IHC, immunofluorescence, and tissue manganese content detection

Data analysis for IHC, immunofluorescence, and tissue manganese content detection was performed using SPSS 26.0 statistical software. All data were first tested for normality using the Shapiro-Wilk test and for homogeneity of variance. If data met both normality and homogeneity of variance, paired-sample t-tests were used, described as mean ± standard deviation (x̄ ± s). If data did not meet normality or homogeneity of variance, the non-parametric Wilcoxon signed-rank test was used, described as M (P25, P75) (M: median, P25: 25th percentile, P75: 75th percentile). *P* < 0.05 was considered statistically significant. All charts were plotted using GraphPad Prism 10.0 software.

## Results

### Proteomics data quality assessment

DIA data were analyzed using Spectronaut 19 software with strict control over identification quality (precursor-level FDR ≤ 1% and protein-level FDR ≤ 1%, requiring at least 2 unique peptides). A high-quality quantitative proteomics dataset was successfully constructed in this study, identifying a total of 10,627 high-confidence proteins. After preprocessing steps including missing value filtering and imputation, a complete matrix containing 10,543 proteins was obtained for downstream statistical analysis. Among these, over 85% of the proteins were supported by no fewer than 2 unique peptides, indicating a high degree of reliability for the vast majority of protein identifications (Table 3).

**Table 3:** Summary of proteomics data output and quality assessment.

Item	Value
Total Identified Proteins	10,627
Number of Proteins for Downstream Analysis	10,543
Proportion of Proteins Supported by ≥ 2 Unique Peptides	> 85%
Precursor-level FDR Control Threshold	≤ 1%
Protein-level FDR Control Threshold	≤ 1%

### Whole proteome data mining

**Fig. 1:**
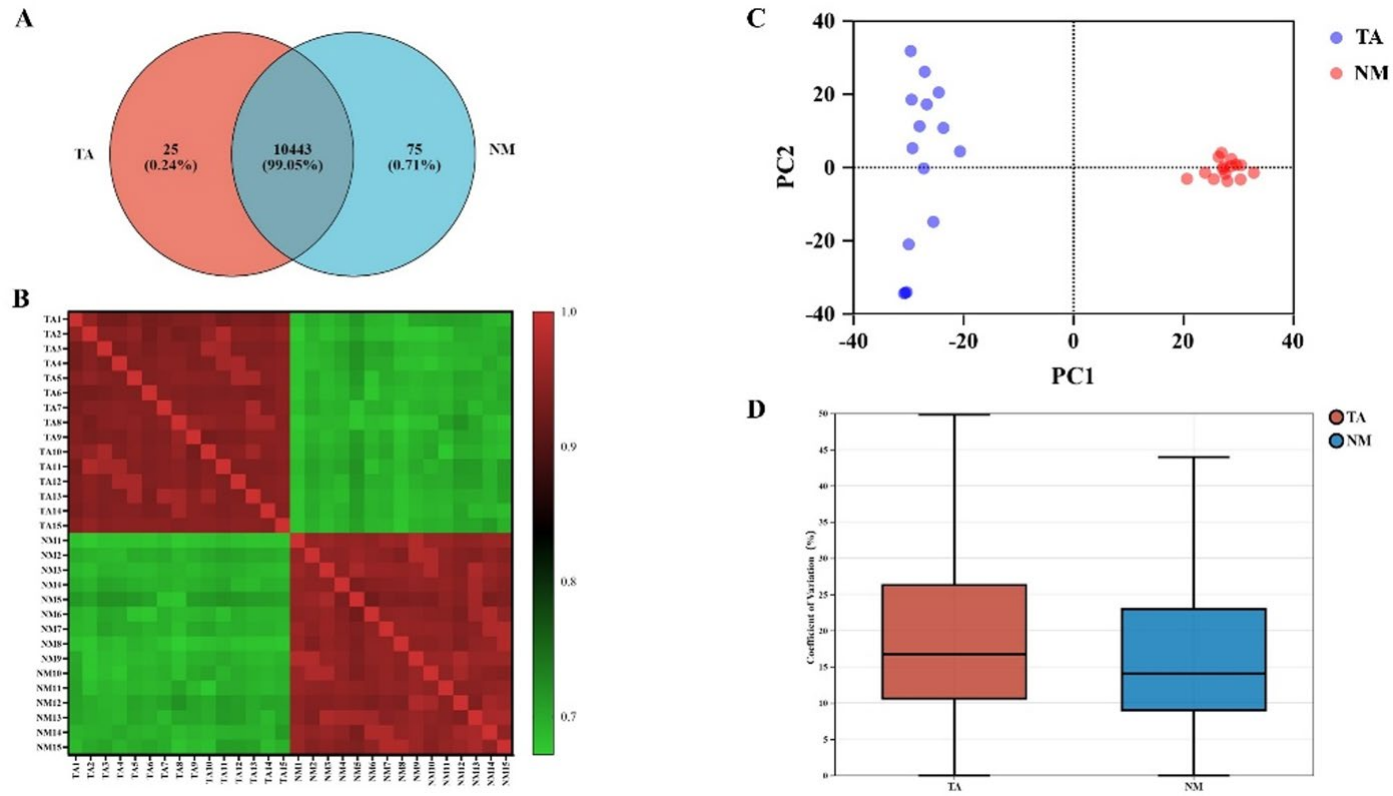
Whole proteome data mining. (**A**) Whole proteome Venn diagram. TA and NM shared 10,443 proteins. (**B**) Sample correlation heatmap. High positive correlations were observed within both TA and NM groups, while correlations between TA and NM samples were lower. (**C**) Principal component analysis (PCA) plot. Significant differences in protein expression existed between TA and NM groups, with samples clustering well within their respective groups. (**D**) Intra-group coefficient of variation (CV) distribution plot. CV values for NM were concentrated in the lower range, suggesting low inter-sample variability; CV values for TA showed a wider distribution and higher mean, indicative of inherent expression heterogeneity among tumor samples; the overall variability levels for both datasets were within acceptable limits.

This study revealed that TA and NM shared 10,443 proteins, accounting for 99.05% (Fig. 1A). The sample correlation heatmap showed significant heterogeneity between TA and NM, but homogeneity within groups, indicating good biological replicates within groups (Fig. 1B). Principal component analysis (PCA) plot showed a clear separation between TA and NM along the PC1 axis, with good intra-group clustering and no overlap between groups, demonstrating significant differences in protein expression between TA and NM (Fig. 1C). The intra-group coefficient of variation (CV) distribution plot showed that CV values for NM were concentrated in the lower range, suggesting low variability among samples; the CV values for TA had a wider distribution and a higher mean, consistent with the heterogeneous nature of adenoma tissues. However, the overall CV levels for both groups were within acceptable limits, indicating the robustness of the quantitative results in this study and supporting subsequent differential analysis (Fig. 1D).

### Differential protein analysis

#### Volcano plot and heatmap

**Fig. 2:**
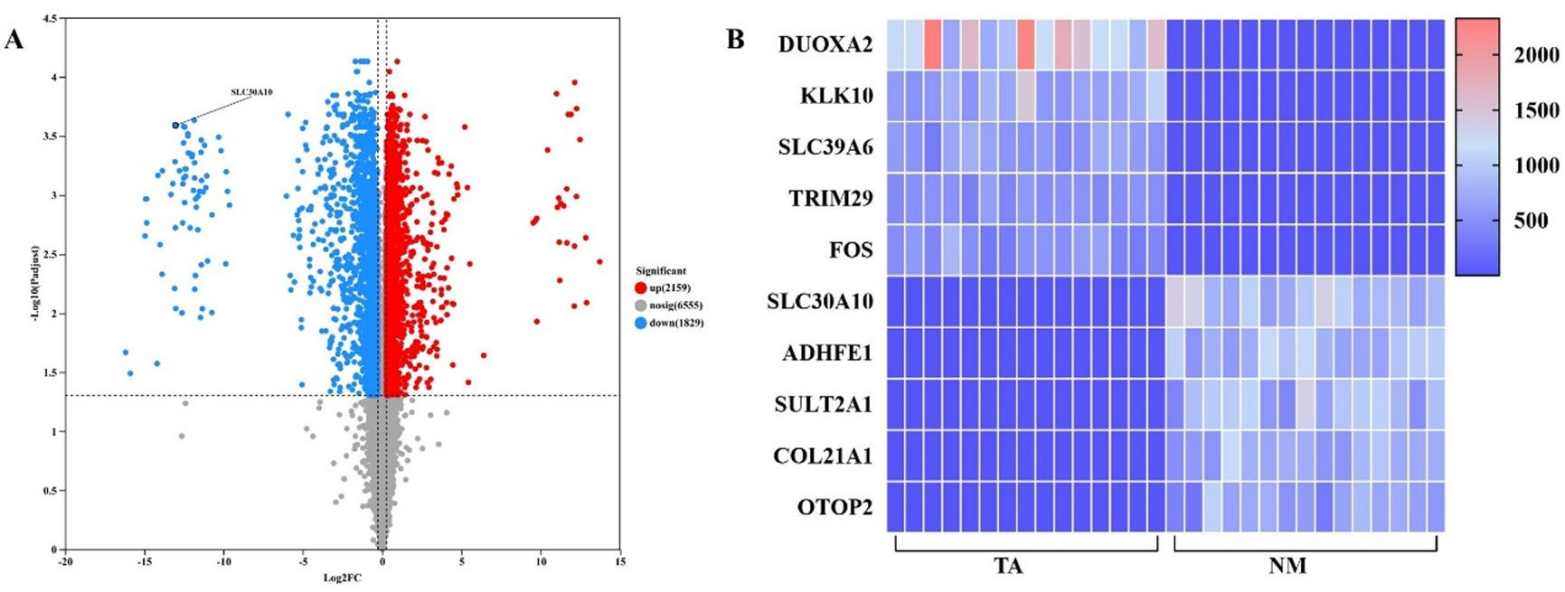
Volcano plot and heatmap of differentially expressed proteins (DEPs). (**A**) Volcano plot. This study identified 2,159 significantly upregulated proteins (red) and 1,829 significantly downregulated proteins (blue). SLC30A10 is indicated on the plot. (**B**) Heatmap. The top 10 most significantly differentially expressed proteins showed significant differences between TA and NM groups.

A total of 3,988 differentially expressed proteins (DEPs) were identified in this experiment, including 2,159 significantly upregulated proteins (red) and 1,829 significantly downregulated proteins (blue). The top 5 proteins with the greatest upregulation were DUOXA2, KLK10, SLC39A6, TRIM29, and FOS, while the top 5 with the greatest downregulation were SLC30A10, ADHFE1, SULT2A1, COL21A1, and OTOP2. A volcano plot (Fig. 2A) and a heatmap (Fig. 2B) were generated based on the data. Details of the DEPs are provided in Appendix 1.

#### GO annotation and enrichment analysis

**Fig. 3:**
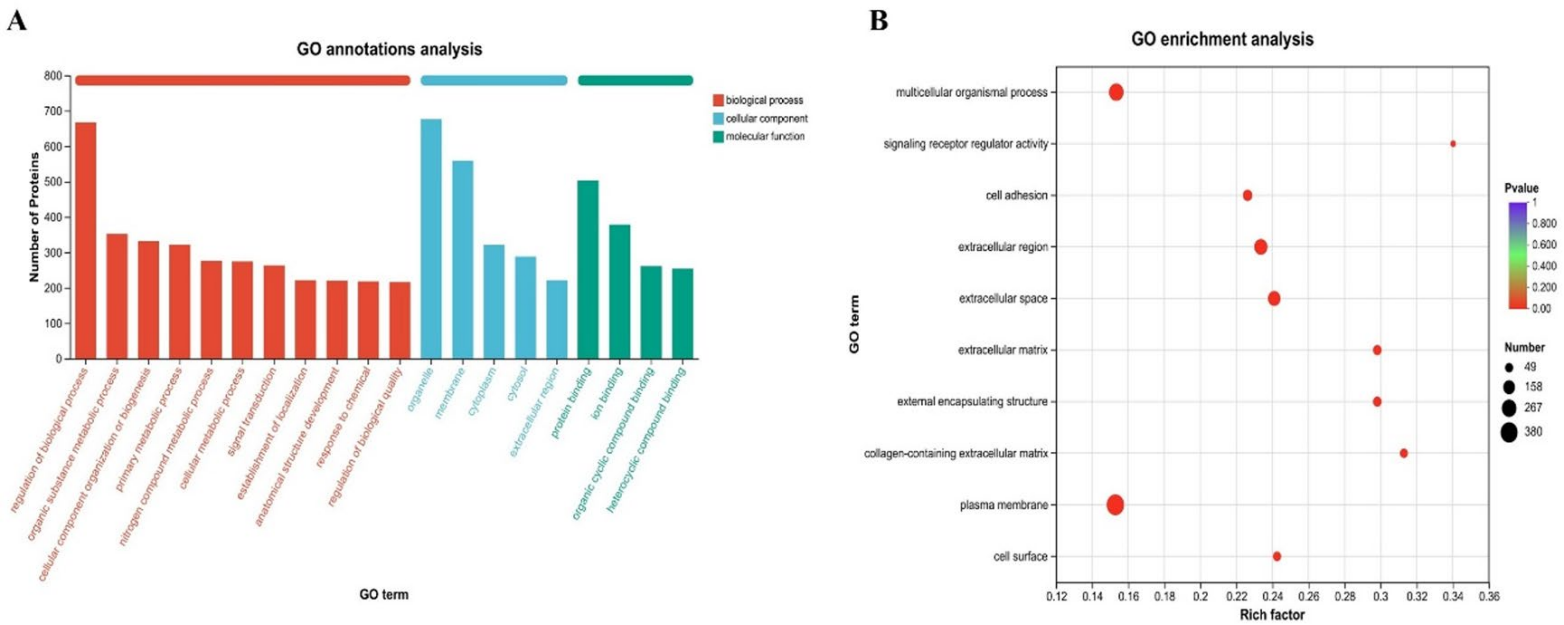
GO annotation and enrichment analysis. (**A**) Annotation analysis. Regarding BP, DEPs were significantly enriched in regulation of biological process and organic substance metabolic process; regarding CC, DEPs were mainly localized to organelles and membranes; regarding MF, DEPs were primarily involved in protein binding and ion binding. (**B**) Enrichment analysis. DEPs were significantly enriched in two major functional modules: signal regulation (signaling receptor regulator activity) and extracellular microenvironment construction (collagen-containing extracellular matrix, extracellular matrix).

To systematically analyze the potential functions of the DEPs, GO annotation analysis was performed. GO annotation comprehensively describes protein attributes from three aspects: Biological Process (BP), Cellular Component (CC), and Molecular Function (MF). The results showed that, regarding BP, the DEPs were significantly enriched in processes such as regulation of biological process and organic substance metabolic process; regarding CC, the DEPs were mainly localized to organelles and membrane structures; regarding MF, the DEPs were primarily involved in protein binding and ion binding (Fig. 3A).

To further identify specific functional modules highly concentrated within the DEP set, GO enrichment analysis was conducted, and the top 10 most significantly enriched terms were visualized. The results indicated that the DEPs were mainly enriched in two broad categories: signal regulation and extracellular microenvironment construction. The former included signaling receptor regulator activity and signal transduction, while the latter included collagen-containing extracellular matrix, extracellular matrix, and external encapsulating structure (Fig. 3B).

#### KEGG pathway annotation and enrichment analysis

**Fig. 4:**
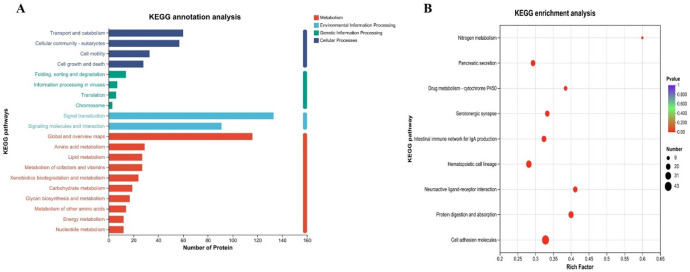
KEGG pathway annotation and enrichment analysis. (**A**) Pathway annotation. DEPs were widely involved in multiple core biological processes, mainly enriched in pathway categories such as Signal transduction, Global and overview maps, Signaling molecules and interaction, Transport and catabolism, Cellular community - eukaryotes, and Amino acid metabolism. (**B**) Enrichment analysis. Nitrogen metabolism, Neuroactive ligand-receptor interaction, and Protein digestion and absorption were the three most significantly enriched pathways for the DEPs.

To reveal the biological pathways and interaction networks involving the DEPs at a systems level, KEGG pathway annotation and enrichment analysis were performed. KEGG pathway annotation results showed that the DEPs were widely involved in several core biological pathways, mainly covering categories such as Signal transduction, Global and overview maps, Signaling molecules and interaction, Transport and catabolism, Cellular community - eukaryotes, and Amino acid metabolism (Fig. 4A).

To further identify key pathways that were statistically significantly enriched, KEGG enrichment analysis was performed. The results showed that the top three enriched pathways were Nitrogen metabolism, Neuroactive ligand-receptor interaction, and Protein digestion and absorption (Fig. 4B).

### Immunohistochemical analysis

**Fig. 5:**
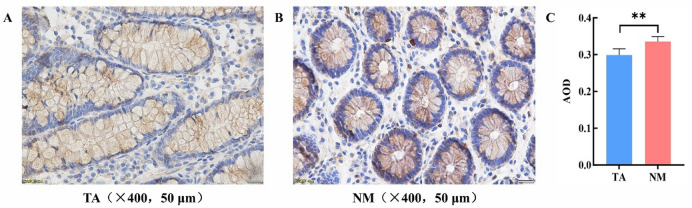
Immunohistochemical detection of SLC30A10 in TA and NM (×400, 50 μm) and its AOD expression (**P < 0.01). (**A**) TA: SLC30A10 shows weak positive membranous and cytoplasmic distribution with distorted glandular architecture. (**B**) NM: SLC30A10 shows moderate positive membranous and cytoplasmic distribution with parallel glandular structures. (**C**) AOD expression: Compared to NM, SLC30A10 expression is significantly decreased in TA.

**Fig. 6:**
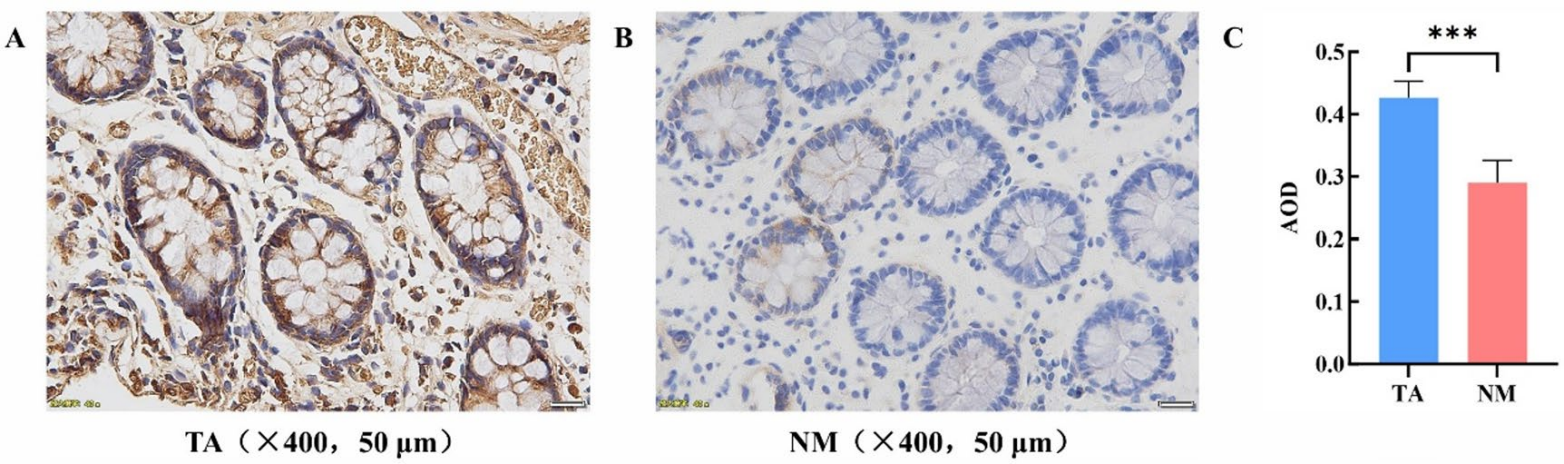
Immunohistochemical detection of cGAS in TA and NM (×400, 50 μm) and its AOD expression (***P < 0.001). (**A**) TA: cGAS shows diffuse moderate positive cytoplasmic distribution; glandular lumens are regular but crowded. (**B**) NM: cGAS shows negative to weak positive distribution; crypts are parallel without crowding. (**C**) AOD expression: Compared to NM, cGAS expression is significantly increased in TA.

**Fig. 7:**
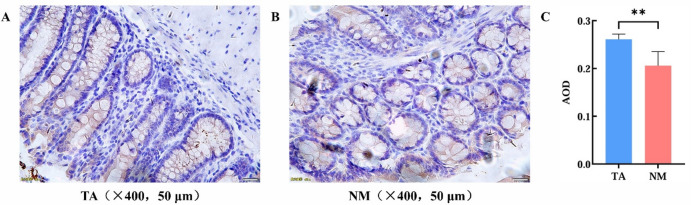
Immunohistochemical detection of STING in TA and NM (×400, 50 μm) and its AOD expression (**P < 0.01). (**A**) TA: STING shows strong positive cytoplasmic and perinuclear aggregation; tubular glands are densely and irregularly arranged. (**B**) NM: STING shows weak positive membranous and cytoplasmic distribution; tubular glands are evenly arranged. (**C**) AOD expression: Compared to NM, STING expression is significantly increased in TA.

**Fig. 8:**
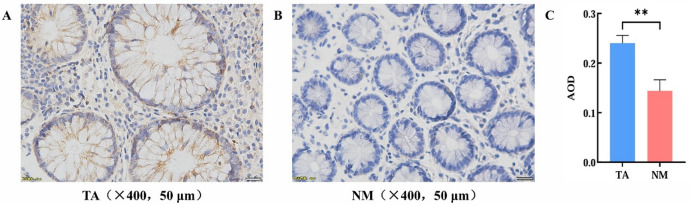
Immunohistochemical detection of p-IRF3 in TA and NM (×400, 50 μm) and its AOD expression (**P < 0.01). (**A**) TA: p-IRF3 shows strong positive nuclear distribution; goblet cell mucous vacuoles are smaller or irregular. (**B**) NM: p-IRF3 shows weak negative distribution; no cellular atypia is observed. (**C**) AOD expression: Compared to NM, p-IRF3 expression is significantly increased in TA.

**Fig. 9:**
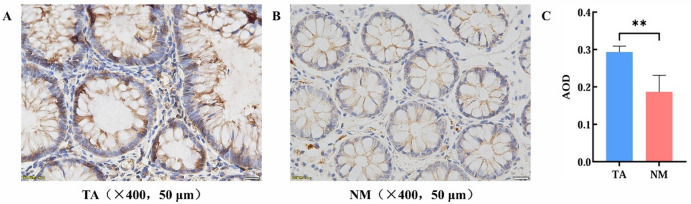
Immunohistochemical detection of ISG15 in TA and NM (×400, 50 μm) and its AOD expression (**P < 0.01). (**A**) TA: ISG15 shows strong positive nuclear and cytoplasmic expression; glandular arrangement is irregular. (**B**) NM: ISG15 shows weak positive membranous and cytoplasmic expression; glands are evenly distributed. (**C**) AOD expression: Compared to NM, ISG15 expression is significantly increased in TA.

**Fig. 10:**
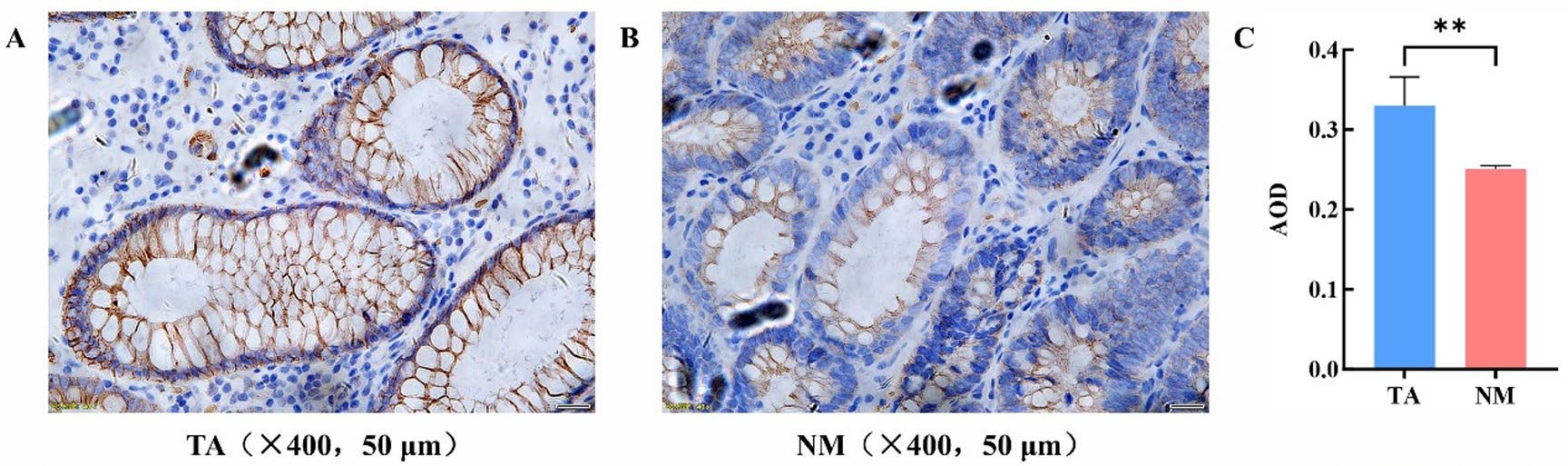
Immunohistochemical detection of β-catenin in TA and NM (×400, 50 μm) and its AOD expression (**P < 0.01). (**A**) TA: β-catenin shows strong positive nuclear and cytoplasmic expression; glands are dense and irregularly branched, with epithelial cells displaying significant atypia. (**B**) NM: β-catenin shows positive membranous and cytoplasmic expression; epithelial nuclei are located at the base and are uniform in size. (C) AOD expression: Compared to NM, β-catenin expression is significantly increased in TA.

Immunohistochemical staining was performed on TA and NM tissues. The results showed that compared to the NM group, the TA group exhibited significant downregulation of SLC30A10 (Fig. 5, **P < 0.01), significant upregulation of cGAS (Fig. 6, ***P < 0.001), significant upregulation of STING (Fig. 7, **P < 0.01), significant upregulation of p-IRF3 (Fig. 8, **P < 0.01), significant upregulation of ISG15 (Fig. 9, **P < 0.01), and significant upregulation of β-catenin (Fig. 10, **P < 0.01). The Average Optical Density (AOD) values for each detected marker are presented in Table 4.

**Table 4:** Expression of AOD for each detected marker in TA and NM (x̄ ± s).

Marker	TA (x̄ ± s)	NM (x̄ ± s)
SLC30A10	0.2988 ± 0.0171**	0.3356 ± 0.0135
cGAS	0.4264 ± 0.0266***	0.2904 ± 0.0355
STING	0.2610 ± 0.0110**	0.2060 ± 0.0294
p-IRF3	0.2404 ± 0.0154**	0.1439 ± 0.0224
ISG15	0.2933 ± 0.0158**	0.1870 ± 0.0443
β-catenin	0.3298 ± 0.0360**	0.2505 ± 0.0043

### Immunofluorescence double staining analysis

**Fig. 11:**
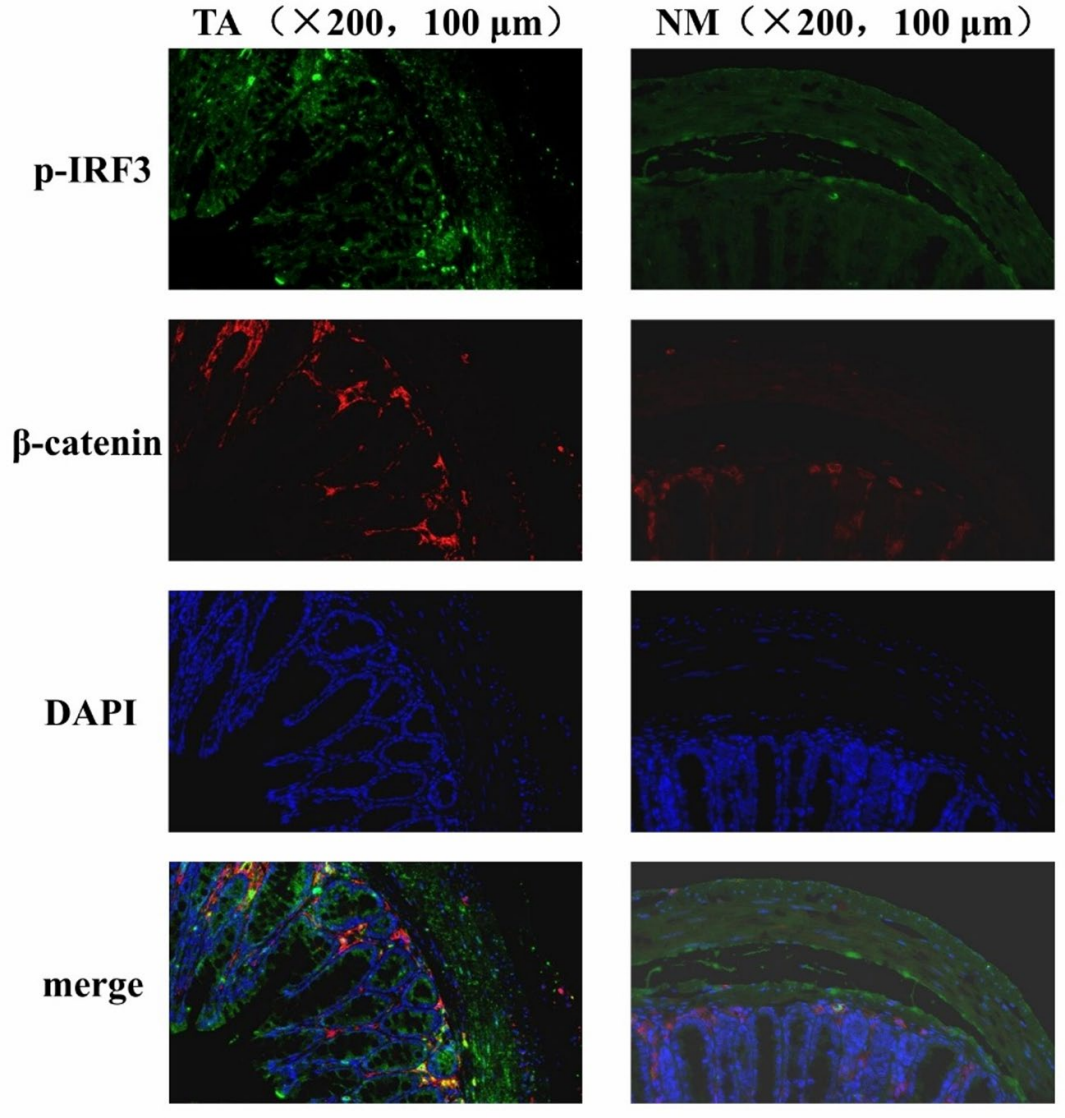
Immunofluorescence double staining for β-catenin and p-IRF3 (×200, 100 μm). Compared to NM, the signal intensity of p-IRF3 and β-catenin is markedly enhanced in TA colonic epithelial cells. The merged image shows co-localization of p-IRF3 and β-catenin in some TA cells.

Immunofluorescence double staining for β-catenin (red) and p-IRF3 (green) was performed on TA and NM tissues. The results showed that compared to NM, the signal intensity of both p-IRF3 and β-catenin was markedly enhanced in TA colonic epithelial cells. Furthermore, TA colonic epithelial cells exhibited nuclear accumulation of β-catenin, whereas in NM, β-catenin was mainly localized in the cytoplasm. The merged images further revealed co-localization of p-IRF3 and β-catenin in the nucleus of some TA cells, suggesting a potential spatial association that warrants further investigation (Fig. 11).

### Tissue manganese content detection

**Fig. 12:**
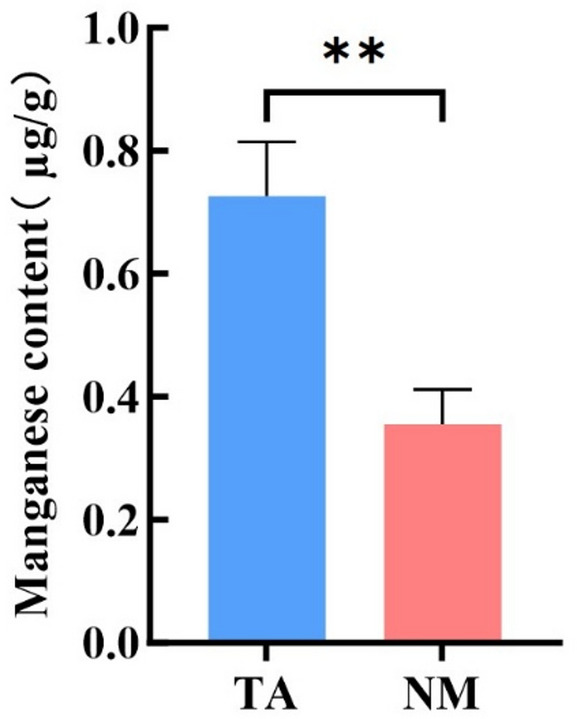
Determination of tissue manganese content in TA and NM. Compared to NM, the tissue manganese content is significantly increased in TA (**P < 0.01).

Inductively coupled plasma mass spectrometry (ICP-MS) analysis revealed that the tissue manganese content in TA was 0.7266 ± 0.0882 µg/g, which was significantly higher than that in NM (0.3551 ± 0.0567 µg/g), with a statistically significant difference (**P < 0.01) (Table 5; Fig. 12).

**Table 5:** Tissue manganese content in TA and NM (µg/g).

Marker	TA (x̄ ± s)	NM (x̄ ± s)
Tissue manganese content	0.7266 ± 0.0882**	0.3551 ± 0.0567

## Discussion

Through DIA proteomic analysis of TA and NM, we found that the intestinal mucosa largely maintains its basal protein expression profile during tubular adenoma formation, yet significant heterogeneity in protein expression exists between the two groups. The sample correlation heatmap showed high correlation within the TA, a significantly lower correlation within the NM, and clear separation of the two groups by hierarchical clustering, revealing the high homogeneity of protein expression in TA and its biological heterogeneity from NM. The volcano plot of DEPs showed a gradient distribution along the vertical axis, suggesting a hierarchical functional regulatory network among DEPs with varying significance levels.

GO functional annotation of the DEPs indicated that tubular adenoma tissues were significantly involved in regulation of biological process and organic substance metabolic process within the BP category, suggesting widespread regulatory network disruption and metabolic reprogramming in TA. Enrichment in the CC category focused on organelles and membrane structures, possibly reflecting abnormalities in intracellular membrane trafficking or surface receptor function. The MF category was dominated by protein binding and ion binding, implying that dysregulation of protein interaction networks and ion homeostasis might be underlying driving factors^8^. GO enrichment analysis revealed significant enrichment of receptor ligand activity and signaling receptor regulator activity, pointing to aberrant ligand-receptor interactions in the adenoma microenvironment. Enrichment of the collagen-containing extracellular matrix indicates exacerbated matrix remodeling, a characteristic feature of precancerous microenvironments, potentially related to tumor invasion and immune evasion^9,10^. These findings are consistent with known mechanisms of CRC development, where dysregulation of receptor-signaling pathways can drive adenoma proliferation, and increased collagen deposition in the extracellular matrix promotes stromal fibrosis, both synergistically accelerating the malignant transformation of adenomas.

KEGG pathway annotation of the DEPs showed that intestinal tubular adenoma tissues were significantly enriched in pathways related to signal transduction and signaling molecules and interaction, indicating widespread dysregulation of signaling networks in adenoma cells. Concurrently, the activity of amino acid and lipid metabolism pathways suggests that metabolic reprogramming may be a core mechanism for energy supply in adenomas^11^. KEGG enrichment analysis indicated significant enrichment of the Nitrogen metabolism pathway, providing a nitrogen source for the rapid proliferation of adenoma cells. Abnormalities in the Neuroactive ligand-receptor interaction pathway suggest disordered intestinal neuroendocrine regulation, which may drive abnormal secretion and proliferation in adenomas. Enrichment of the Protein digestion and absorption pathway reflects enhanced degradation of the extracellular matrix, promoting microenvironment remodeling and tumor invasion. These three elements together constitute a pathological axis of “metabolism-signaling-microenvironment,” facilitating adenoma formation and carcinogenesis^12^.

SLC30A10 is a manganese homeostasis regulatory protein primarily localized on the cell membrane. It is expressed on the canalicular membrane of hepatocytes and the apical membrane of intestinal epithelial cells. By mediating manganese efflux, it maintains systemic manganese homeostasis, preventing neurotoxicity and liver damage caused by manganese accumulation. In colorectal adenocarcinoma tissues, mRNA and protein levels of SLC30A10 are generally downregulated^13^. Taylor Cherish’s team found that liver- and intestine-specific knockout mice exhibited significantly elevated manganese levels in the brain, blood, and liver, demonstrating that SLC30A10 in the digestive system is a core regulator of basal manganese homeostasis. Intestinal epithelial-specific SLC30A10 knockout mice developed manganese accumulation in the colonic epithelium, cellular hyperproliferation, and early adenoma-like changes^14^. Other studies have shown that knockdown of SLC30A10 in colorectal adenocarcinoma cell lines such as HCT-15, SW480, and HuTu80 leads to manganese accumulation, subsequently promoting cancer cell proliferation and migration, whereas SLC30A10 overexpression has the opposite effect^15^. Previous research suggests a protective role for SLC30A10 in colorectal adenocarcinoma. The current study found that SLC30A10 is significantly downregulated as early as the tubular adenoma stage, potentially offering new avenues for the diagnosis and treatment of the “adenoma-carcinoma” sequence.

The cGAS-STING pathway is a core target in the field of tumor immunotherapy in recent years. In early stages, it can promote type I interferon production and CD8⁺ T cell infiltration, delaying the transformation of adenoma to adenocarcinoma. However, persistent STING agonism can promote IRF3 phosphorylation and nuclear translocation, accelerating tumor progression and drug resistance^16^. Other studies indicate that IRF3 acts as a negative regulator. Under normal conditions, it can bind and sequester β-catenin in the cytoplasm, preventing its nuclear entry and thereby inhibiting the activation of the Wnt/β-catenin signaling pathway. When the cGAS-STING pathway is persistently activated, nuclear p-IRF3 may release its sequestration of β-catenin, promoting β-catenin nuclear translocation and subsequently enhancing Wnt signaling pathway activity^17^. Based on existing research evidence, our team proposes the following mechanistic hypothesis: During the development of colorectal adenoma, downregulation of SLC30A10 expression leads to intracellular manganese accumulation. Manganese may directly activate cGAS, promoting the synthesis of the second messenger cGAMP and activating STING. STING recruits and mediates the phosphorylation of IRF3, facilitating p-IRF3 nuclear entry. Once in the nucleus, p-IRF3 releases its cytoplasmic sequestration of β-catenin, leading to increased β-catenin nuclear translocation, thereby activating the canonical Wnt/β-catenin signaling pathway and accelerating the initiation and progression of colorectal adenoma.

Based on the above hypothesis, we conducted preliminary validation through immunohistochemistry, immunofluorescence double staining, and tissue manganese content measurement. The results showed that compared to NM, the expression of SLC30A10 was significantly decreased in TA, consistent with the initial DIA findings, suggesting that functional downregulation of SLC30A10 may already exist at the TA stage. Concurrent with SLC30A10 downregulation, manganese content in TA tissues was significantly elevated, confirming an imbalance in manganese homeostasis accompanies this process. Meanwhile, key molecules of the cGAS-STING pathway—cGAS, STING, and their downstream effectors p-IRF3 and ISG15—were all significantly upregulated in TA. Immunofluorescence double staining further showed enhanced nuclear signals of p-IRF3 in TA cells and spatial co-localization of p-IRF3 with nuclear β-catenin. These findings suggest potential associations among SLC30A10 downregulation, increased tissue manganese content, upregulation of cGAS-STING pathway-related molecules, and β-catenin nuclear translocation in colorectal tubular adenoma. We speculate that SLC30A10 downregulation may cause manganese accumulation, which in turn might be related to the upregulation of cGAS-STING pathway-related molecules. Furthermore, immunofluorescence double staining revealed co‑localization of p-IRF3 and β-catenin in the nucleus of some adenoma cells. Based on this observation and previous reports^16,17^, it is possible that nuclear p-IRF3 may influence β-catenin subcellular distribution; however, the precise relationship requires further experimental validation.

However, the current findings are primarily correlative. Causal relationships among the involved proteins, whether manganese ions directly activate cGAS, and how p-IRF3 specifically influences β-catenin subcellular localization require further validation using molecular biology approaches such as knockdown/overexpression, chromatin immunoprecipitation, and luciferase reporter assays in cellular models. In addition, although SLC30A10 and STING showed statistically consistent expression trends in paired samples and were both predominantly localized in the glandular epithelium, their co‑localization at the cellular level was not verified by double immunofluorescence on the same or matched serial sections. Further studies using matched serial sections or double staining are warranted to clarify their spatial relationship, and expansion of the clinical sample size together with intestinal epithelial‑specific SLC30A10 knockout mouse models would help systematically evaluate the overall role of this axis in adenoma development and its potential as an early intervention target.

## Conclusion

Through DIA proteomics and multi-omics validation analysis, this study preliminarily explored potential molecular events in the development of colorectal tubular adenoma. The research found that SLC30A10 is significantly downregulated in TA tissues, accompanied by an increase in tissue manganese content, suggesting that manganese homeostasis imbalance may participate in the early stages of adenoma progression. Functional enrichment analysis supports the existence of widespread signal transduction and metabolic reprogramming in adenomas. Further immunohistochemistry and immunofluorescence results showed that concurrent with SLC30A10 downregulation, cGAS, STING, p-IRF3, and β-catenin exhibited an upregulation trend. Moreover, immunofluorescence double staining revealed co-localization of p-IRF3 and β-catenin in the nucleus of some adenoma cells, suggesting a potential spatial association that warrants further mechanistic investigation. In summary, downregulation of SLC30A10 expression may trigger manganese homeostasis disruption, which could subsequently be associated with upregulation of cGAS-STING pathway-related molecules and enhanced Wnt/β-catenin signaling. This may represent a potential link connecting innate immune abnormalities with classical pro-tumorigenic pathways, and its specific mechanisms warrant further experimental validation.

## Supplementary Information

Below is the link to the electronic supplementary material.

Supplementary Material 1

## Data Availability

All data generated or analysed during this study are included in this published article and its supplementary information files.
