# Supplementary material for: SLC30A10 downregulation is associated with cGAS-STING pathway activation in colorectal tubular adenoma
Source: Sci Rep. 2026 Apr 16;16:17717. doi: 10.1038/s41598-026-48815-6 (PMC13246812; doi:10.1038/s41598-026-48815-6)
Supplement: Supplementary file 1 — Supplementary Material 1 [file 41598_2026_48815_MOESM1_ESM.pdf]

Appendix 1: Details of Differentially Expressed Proteins

Symbol	Log2FC(TA/NM)	P_value(TA/NM)	P_adjust(TA/NM)	Regulate	TA1	TA2	TA3	TA4	TA5	TA6
DUOXA2	13.730	0.000605	0.003665	up	1128.899	1196.803	2328.208	696.531	1639.263	723.041
KLK10	12.845	0.000285	0.002289	up	600.971	508.515	554.421	761.168	519.269	803.817
SLC39A6	12.485	6.32E-06	0.000329	up	602.734	527.872	309.400	678.761	754.159	656.944
TRIM29	12.264	7.74E-07	0.000176	up	456.096	499.346	503.594	511.545	355.021	627.178
FOS	12.255	7.35E-05	0.001019	up	475.224	572.634	408.990	826.985	483.413	301.604
SLC30A10	-13.197	4.25E-05	0.000791	down	0.100	0.100	0.100	0.100	0.100	0.100
ADHFE1	-13.062	1.79E-05	0.000512	down	0.100	0.100	0.100	0.100	0.100	0.100
SULT2A1	-13.033	0.000212	0.001881	down	0.100	0.100	0.100	0.100	0.100	0.100
COL21A1	-12.778	2.62E-05	0.000614	down	0.100	0.100	0.100	0.100	0.100	0.100
OTOP2	-12.578	0.000182	0.00171	down	0.100	0.100	0.100	0.100	0.100	0.100

continued

Symbol	TA7	TA8	TA9	TA10	TA11	TA12	TA13	TA14	TA15	NM1	NM2	NM3
DUOXA2	892.295	2264.657	1162.851	1762.505	1512.369	1167.897	1181.152	807.668	1578.476	0.100	0.100	0.100
KLK10	678.797	1459.043	554.743	531.468	657.795	640.219	661.543	741.307	1068.920	0.100	0.100	0.100
SLC39A6	524.839	531.253	565.303	418.636	494.081	716.460	705.551	590.891	528.046	0.100	0.100	0.100
TRIM29	415.057	567.746	477.721	501.470	507.569	433.283	491.099	521.118	491.402	0.100	0.100	0.100
FOS	313.242	527.871	523.929	490.812	617.987	655.199	392.508	307.423	420.557	0.100	0.100	0.100
SLC30A10	0.100	0.100	0.100	0.100	0.100	0.100	0.100	0.100	0.100	1376.715	1322.672	796.038
ADHFE1	0.100	0.100	0.100	0.100	0.100	0.100	0.100	0.100	0.100	1051.244	534.157	762.21
SULT2A1	0.100	0.100	0.100	0.100	0.100	0.100	0.100	0.100	0.100	405.152	904.187	997.411
COL21A1	0.100	0.100	0.100	0.100	0.100	0.100	0.100	0.100	0.100	480.587	564.874	519.41
OTOP2	0.100	0.100	0.100	0.100	0.100	0.100	0.100	0.100	0.100	378.266	255.481	1045.077

continued

Symbol	NM4	NM5	NM6	NM7	NM8	NM9	NM10	NM11	NM12	NM13	NM14	NM15
DUOXA2	0.100	0.100	0.100	0.100	0.100	0.100	0.100	0.100	0.100	0.100	0.100	0.100
KLK10	0.100	0.100	0.100	0.100	0.100	0.100	0.100	0.100	0.100	0.100	0.100	0.100
SLC39A6	0.100	0.100	0.100	0.100	0.100	0.100	0.100	0.100	0.100	0.100	0.100	0.100
TRIM29	0.100	0.100	0.100	0.100	0.100	0.100	0.100	0.100	0.100	0.100	0.100	0.100
FOS	0.100	0.100	0.100	0.100	0.100	0.100	0.100	0.100	0.100	0.100	0.100	0.100
SLC30A10	666.443	1055.053	614.096	704.263	978.802	1349.693	1059.355	731.24	860.748	834.574	659.179	841.532
ADHFE1	594.521	730.035	1131.536	909.984	1128.686	792.701	648.184	678.366	662.278	930.785	1020.76	1019.335
SULT2A1	1010.482	1071.05	556.115	423.924	1339.069	654.67	950.799	1003.947	1040.766	813.582	490.019	881.497
COL21A1	1133.794	757.338	687.325	744.18	728.817	522.73	542.142	826.602	945.566	722.332	715.753	736.498
OTOP2	649.819	700.468	755.14	530.726	577.108	316.873	650.279	847.448	675.144	727.804	642.933	553.917
